# Neuroendocrine Factors and Head and Neck Squamous Cell Carcinoma: An Affair to Remember

**DOI:** 10.1155/2018/9787831

**Published:** 2018-05-08

**Authors:** Iulia Solomon, Vlad Mihai Voiculescu, Constantin Caruntu, Mihai Lupu, Alexandra Popa, Mihaela Adriana Ilie, Radu Albulescu, Ana Caruntu, Cristiana Tanase, Carolina Constantin, Monica Neagu, Daniel Boda

**Affiliations:** ^1^Department of Dermatology and Allergology, Elias Emergency University Hospital, Bucharest, Romania; ^2^Department of Dermatology, Carol Davila University of Medicine and Pharmacy, Bucharest, Romania; ^3^Department of Physiology, Carol Davila University of Medicine and Pharmacy, Bucharest, Romania; ^4^Department of Dermatology, “Prof. N. C. Paulescu” National Institute of Diabetes, Nutrition and Metabolic Diseases, Bucharest, Romania; ^5^Department of Dermatology, MEDAS Titan Medical Center, Bucharest, Romania; ^6^Dermatology Research Laboratory, Carol Davila University of Medicine and Pharmacy, Bucharest, Romania; ^7^Department of Biochemistry, Carol Davila University of Medicine and Pharmacy, Bucharest, Romania; ^8^Chemical and Pharmaceutical National Institute, Bucharest, Romania; ^9^Department of Oral and Maxillofacial Surgery, Carol Davila Central Military Emergency Hospital, Bucharest, Romania; ^10^Faculty of Medicine, Titu Maiorescu University, Bucharest, Romania; ^11^Victor Babes National Institute of Pathology, Bucharest, Romania; ^12^Colentina Clinical Hospital, Bucharest, Romania; ^13^Faculty of Biology, University of Bucharest, Bucharest, Romania

## Abstract

Head and neck squamous cell carcinoma (HNSCC) is one of the most aggressive malignancies. Therefore, the major goal of cancer treatment is inhibition of tumor cell growth and of metastasis development. In order to choose the best management option for HNSCC patients, we need to identify reliable prognostic factors and to develop new molecular techniques in order to obtain a better understanding of therapy resistance. By acting as neurohormones, neurotransmitters, or neuromodulators, the neuroendocrine factors are able to signal the maintenance of physiological homeostasis or progression to malignant disease. Certain neuropeptides possess strong antitumor properties acting as tumor suppressors and immunomodulators, providing additional benefits for future potential therapeutic strategies. In light of the current understanding, cancer starts as a localized disease that can be effectively treated if discovered on proper time. Unfortunately, more than often cancer cells migrate to the surrounding tissues generating distant metastases, thus making the prognosis and survival in this stage much worse. As cellular migration is mandatory for tumor invasion and metastasis development, searching for alternate controllers of these processes, such as the neuroendocrine factors, it is an active tremendous task.

## 1. Introduction

As the sixth most common cancer worldwide, head and neck squamous cell carcinoma (HNSCC) is one of the most aggressive malignancies. Since most of the patients diagnosed with HNSCC have metastases at the time of their initial examination, it is well known that the surviving rate is very low and the prognosis is even worse than other sold cancers like melanoma or breast cancer [1–4].

Surgery—as the eventual therapeutic option—often compromises essential functions such as speech and swallowing, which considerably impair the quality of life. However, excision of the tumor is sometimes inadequate, as more than 90% of cancer deaths do not originate from the primary tumor, but from the development of metastases. Thus, for a more systematic approach, chemotherapy has been heavily involved in destroying tumor cells leading to great success in cancer treatment over the past decades. Yet, there are tumor cells that are not affected by the chemotherapy still resulting in tumor progression and metastasis. Therefore, the major goal of the cancer treatment is inhibition of tumor cell growth and of metastasis development. In order to choose the best management option for HNSCC patients, we need to identify reliable prognostic factors and to develop new molecular techniques in order to obtain a better understanding of therapy resistance. Serpentine receptor ligands, chemokines, and neurotransmitters have been extensively studied in recent years to find new therapeutic targets in HNSCC [5–7].

As one of the most important events controlling the release of cytokines, inflammation has often been directly involved in tumor development, migration, and progression [8–11]. Moreover, some studies have highlighted that tumor cells use chemokine gradients to spread in different anatomic sites of the body [12].

Ever since the first decades of the previous century, research has highlighted the implication of psychosocial factors and of neurotransmitters and hormones as components of the neuroendocrine system, in the occurrence and progression of cancer [13]. Numerous studies have presented neurotransmitters as the key factors in regulating tumor cell migration. [14]. Hence, recent advances in molecular biology have led to new diagnostic and therapeutic strategies [15–19]. Although less advanced than breast, renal, or colorectal cancer treatments, HNSCC therapy is in constant evolution [20, 21].

Potential clinical applications are promising because both chemokines and neurotransmitters are ligands to serpentine receptors, and it is important to emphasize that several chemokines can bind to one receptor which means that blocking one receptor would lead to the inhibition of several chemokine functions, resulting a possible deregulation of the immune system. In contrast, in the case of neurotransmitters, a ligand can bind to several tissue-specific receptors. Thus, a receptor could be inhibited without affecting the neurotransmitter function as a whole [8].

The migration of breast or colon cancer cells can be inhibited by specific or nonspecific adrenergic blockers [22, 23]. Numerous studies have come to support this theory. Selective antagonists for several neurotransmitters are already available and in widespread clinical use for other pathologies, for example, *β*-blockers for the treatment of cardiovascular diseases. For instance, it was found that patients treated for arterial hypertension with *β*-blockers had significantly lower mortality rates [8].

Nevertheless, it is known that *β*-blockers are not commonly used as a general cancer prevention treatment because they are selectively effective only in cancers stimulated by *β*-adrenergic agonists. Moreover, it is also recommended to prescreen the patients for the detection of stress neurotransmitter levels and cAMP concentrations. It should be emphasized that *β*-blockers are sometimes contraindicated because they can promote certain types of cancer due to the fact that cAMP acts as a tumor promoter in some cancers, and tumor suppressor in others [24].

**Figure 1:**
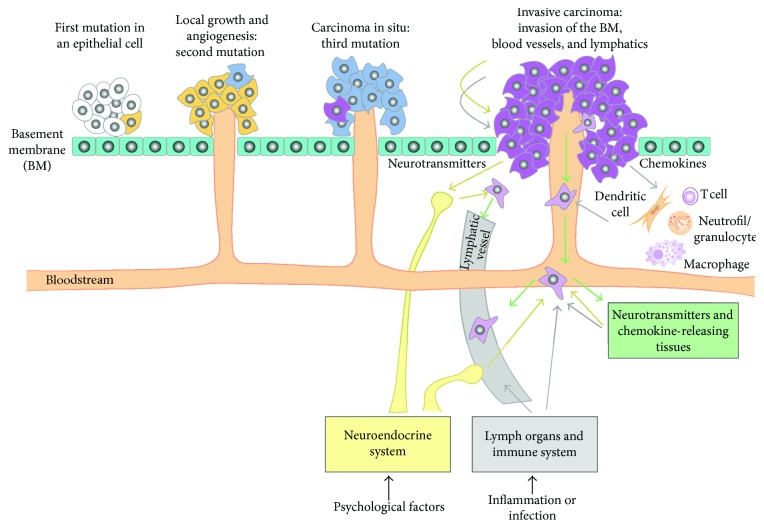
Chronology of cellular events occurring during tumor progression and regulation by chemokines and neurotransmitters of metastasis formation. Cell migration is initiated in the primary tumor by chemokines (grey arrows) and neurotransmitters (yellow arrows). This further leads to dissemination via hematogenous or lymphatic routes. Finally, tumor cells migrate towards a source of chemokines and neurotransmitters.

In the medical literature, neurotransmitters are quoted as being actively involved in tumor invasion and metastasis, analogous to chemokines. Furthermore, the neuropeptide innervation of the tumor has supported this hypothesis. In addition, since tumor cell migration and multiplication is an essential condition for invasion and metastasis, tumor biology has directed its studies to identify the factors that regulate the migration of these cells which is dependent on signal biomolecules of the immune and neuroendocrine systems (see Figure 1). This functional interaction is an essential breakthrough in metastatic development and progression as it proposes new pathways for specific inhibition of tumor cell invasion and metastasis. Research on how all these receptors regulate the activity of specific antagonists could suggest new therapeutic means to avoid tumor invasion and metastasis [25]. Thus, they may lay the foundation for studies that investigate new pathways for specific inhibition of tumor cells and metastases.

By acting as neurohormones, neurotransmitters, or neuromodulators, the neuroendocrine factors are able to signal the maintenance of physiological homeostasis or progression to malignant disease. Epigenetic changes are essential for regulating gene expression and for controlling cancer progression, these being hereditary without causing changes in the DNA sequence. Although there are numerous studies on the epigenetic regulation of neuropeptides, they are part of a field that is still under development [26].

Certain neuropeptides possess strong antitumor properties, tumor suppressors, and immunomodulators, providing additional benefits for future potential therapeutic strategies. However, it has been shown that certain neuropeptides such as substance P (SP) possess strong procarcinogenic properties and can stimulate the migration of tumor cells. In various tumors, SP is synthesized and secreted by both tumor and nontumor cells and may act as a mitogen factor via NK-1 receptor expressed by tumor cells [27–36].

In light of the current understanding, cancer starts as a localized disease that can be effectively treated if discovered on proper time. Unfortunately, more than often cancer cells migrate to the surrounding tissues generating distant metastases, thus making the prognosis and survival in this stage much worse. As cellular migration is mandatory for tumor invasion and metastasis development, searching for alternate controllers of these processes, such as the neuroendocrine factors, it is an active tremendous task.

## 2. Role of Stress-Related Neuroendocrine Factors

Although hard to define or measure, stress has been shown to be involved in the development and progression of cancer through the hormonal and immune changes it produces. Stress-induced activation of the sympathetic nervous system and hypothalamic-pituitary-adrenal (HPA) axis leads to a significant increase in cortisol, norepinephrine (NE), and epinephrine (E) levels [37–43]. Stress hormones have the ability to act directly or indirectly on tumor cells by regulating the production of cytokines, chemokines, and growth factors [37, 39, 44].

Imbalances of neuroendocrine factors, such as those associated with the stress response of the body, may be involved in modulating of the carcinogenesis process. These can accelerate the appearance of cutaneous-mucosal tumors and their progression as well as suppress tumor regression. Thus, animal studies have shown that chronic stress accelerates the process of developing of SCC and also stimulates tumor progression and inhibits tumor regression. These effects appear to be induced by reduction of T cell-mediated immune response. Chronic stress exposure reduces the number of CD4^+^ and CD8^+^ cells that infiltrate the peritumoral region, at the same time increasing the number of CD25^+^ cells. Chronic stress was also associated with reduction of IFN-*γ* and IL-12, cytokines with a well-known antitumoral effect [45] On the other hand, acute stress appears to have a protective effect in experimental animals, short-term exposure to stressors being associated with a lower risk of developing SCC and a decrease in the number of tumors. It also induces higher levels of IL-12 and IFN-*γ*, an increased number and a stronger activity of CD8^+^ and CD4^+^ T cells in the skin [46].

The involvement of the hormones associated with corticotropin-releasing hormone (CRH)-proopiomelanocortin (POMC) axis in the tumor development process has also prompted the interest of researchers. Thus, a study that performed an immunohistochemical analysis of the expression of these hormones in biopsy specimens from cutaneous tumors revealed that 70% of the SCC are highly immunoreactive for CRH, 80% for ACTH, and 60% for *α*-MSH [47].

Exposure to a cortisol concentration similar to that of stress conditions induced an increase in SCC15 cell proliferation and resulted in a slight increase in IL-6 expression in SCC9, SCC15, and SCC25 cells. However, at higher concentrations, it induced a decrease of IL-6 release in SCC9 and SCC15 cells, suggesting a possible dual and concentration-dependent role of cortisol [48]. Hence, a dual role of cortisol is assumed: firstly, a proinflammatory role in physiological stress and, on the other hand, a role of reducing the levels of proinflammatory cytokines in pharmacological doses. However, the influence of cortisol, as well as other stress hormones on HNSCC, has been scarcely investigated compared to other types of cancer [49], and previous studies have not revealed notable effects of glucocorticoid hormones on the proliferation of HNSCC [50].

Conditions such as depression, anxiety, and chronic stress can lead to an imbalance of the HPA axis [51, 52] and an increase of proinflammatory cytokines such as IL-6 [53]. They are also associated with an increase of vascular endothelial growth factor (VEGF) production [54]. VEGF is one of the most important angiogenesis stimulators and it has long been demonstrated to facilitate tumor development and metastasis. Studies have shown that high levels of VEGF in HNSCC patients are correlated with poorer prognosis and a decreased survival rate [55, 56]. Very important are the studies that investigated the connection between psychosocial impairment and VEGF expression level and implicitly the angiogenesis required both for tumor invasion and its metastasis [57, 58]. Recent research has revealed that HNSCC patients with poor psychosocial functioning and strong VEGF expression have a survival rate significantly lower than what is called the best predictor of survival [59]. Moreover, since depression is common in oral SCC patients, overexpression of IL-6 has been shown to lead to a poorer prognosis of the tumor [60, 61]. Further studies are needed to clarify the mechanisms underlying the tissue and systemic variations of cortisol under stress conditions and their connections with angiogenesis stimulators and proinflammatory cytokines.

Besides VEGF and proinflammatory cytokines, stress-related neuroendocrine factors are able to modulate the expression of matrix metalloproteinases (MMPs). Both MMPs and metalloproteinase tissue inhibitors (TIMP) are involved in remodeling the extracellular matrix in both physiological and pathological processes, being controlled by several cytokines [62]. Previous research has shown a significant increase in MMP9 and MMP2 expression in SCC as compared to BCC, subsequently associated with an increased aggressiveness [63]. It has been found that activation of HPA axis and sympathoadrenomedullary system induces various modifications of MMP levels through cortisol and catecholamine actions [64].

Norepinephrine (NE) and epinephrine (E) are the most documented stress-related catecholamines. The sympathoadrenomedullary system is responsible for variations of both NE and E levels during stress. NE and E activate *α*- and *β*-adrenoreceptors (ARs), being involved in stress-induced tumor progression [65].

The expression of *β*-ARs in human oral SCC, as well as several SCC cell lines, such as SCC9, SCC15, SCC25 [48], and TCa8113 [66], has been previously demonstrated. Furthermore, Shang et al. [66] have revealed that NE stimulates the proliferation of TCa8113 cells, indicating that adrenergic receptors may play an important role in modulation of mechanisms involved in development of this type of cancer.

A complex *in vitro* study [48] investigated the effect of stress hormones such as cortisol and NE but also other adrenergic receptor agonists on proliferation of oral SCC cells and IL-6 secretion. It is worth to point out that IL-6 is a cytokine important in angiogenesis and tumor progression [67], and its levels are increased in both blood and saliva of patients diagnosed with HNSCC [68, 69]. NE induced an increase in SCC9 and SCC15 cells' proliferation, the stimulatory effect being diminished by administration of neutralizing antibodies against IL-6. Also, NE and isoproterenol produced a significant increase of IL-6 release by SCC9 and SCC25 cells, and the connection of *β*-ARs to this mechanism was evidenced by inhibition of these activating effects by *β*-AR antagonists [58].

Stress, as well as direct activation of *β*-ARs, was associated with a significant increase of tumor vascularization, while *β*-blockers reduced the vessel density [70, 71]. In previous studies, it was revealed that tumors associated with stressful conditions have elevated levels of VEGF and other angiogenic factors, such as IL-8 and IL-6 [70]. These are correlated with elevated levels of cAMP that further lead to activation of protein kinase A (PKA) and Src kinase [70, 72]. Also, adrenergic stimulation has been shown to increase the expression of MMPs, such as MMP-2 and MMP-9, thereby promoting the angiogenic and metastatic processes [73].

Dopamine (DA), another biogenic amine expressed under stress, is also an important neurotransmitter in the brain that acts through two types of receptors—D1 and D2, which appear to have an opposite effect than NE and E on tumor growth. DA administration has been shown to inhibit the growth of various tumors by blocking proliferation, migration, and vascular permeability induced by VEGF [74, 75]. Furthermore, DA interferes with VEGF signaling by reducing phosphorylation of VEGF-R2 and preventing activation of kinases from the downstream—focal adhesion kinase (FAK) and p42/44 mitogen-activated protein kinase (MAPK) [76, 77]. Thus, DA receptor agonists could become attractive antiangiogenic drugs in cancer therapy.

Endothelins (ET) are vasoconstricting peptides that were previously linked to stress reaction [78, 79]. ET can be released not only from endothelial cells but also from macrophages, neurons, smooth muscle cells, and oral epithelial cells. There are three isoforms—ET-1, ET-2, and ET-3 [4, 80], but it has been shown that mainly ET-1 is involved in growth and progression of primary tumors and metastases in a variety of cancers, including HNSCC and melanoma [4, 81]. Assessment of the salivary levels of ET-1 in HNSCC may allow identification of high-risk patients for developing aggressive SCC. Moreover, antagonists of ET receptors could represent an adjuvant therapy for these patients [4].

Substance P (SP) is part of the neurokinin family and is strongly expressed in central and peripheral nervous systems in conditions such as stress, anxiety, and depression [82]. SP is an inflammatory molecule, member of the tachykinin neuropeptide family [83], acting via the neurokinin-1 receptor (NK-1R). It plays a central role in the neurogenic inflammatory reaction. Among many cellular effects, the chemotaxis, secretion of proinflammatory cytokines, such as IL-1, IL-6, and TNF-*α*, induction of lymphocyte proliferation, immunoglobulin production, and macrophage activation should be highlighted [84]. Furthermore, SP has a strong mitogenic effect in tumors expressing NK-1R such as melanoma, glioma, retinoblastoma, and neuroblastoma [85, 86]. Previous research on human epithelial cells has shown that binding of SP to NK-1R stimulates intracellular signaling through protein kinases 1 and 2 pathways, leading to protection against apoptosis and to cell proliferation [87]. Moreover, overexpression of SP and NK-1R is correlated with development and progression of oral SCC [88, 89].

A case-control study performed on 90 SCC tumors excised from 73 patients revealed immunohistochemical expression of SP in tumor cell cytoplasm in 81.3% of cases, NK1-R being found in 14% of cases on tumor cell membrane, in 48.3% of cases in infiltrating lymphocytes, and in 22.5% of cases in tumor blood vessels. These results suggest the involvement of SP and NK-1R in the processes of carcinogenesis in oral mucosa, suggesting the importance of deepening research in order to identify new pathophysiological mechanisms and new potential therapeutic targets for the management of patients with oral cancer [89].

In another study, it was evaluated by immunohistochemistry the expression of SP and NK-1R in oral SCC and adjacent nontumoral epithelium. It was investigated also the relationship of this expression with the presence of dysplasia [90]. Nuclear and cytoplasmic expression of SP in the nontumoral epithelium was significantly associated with the presence of epithelial dysplasia and carcinoma in situ. These results suggest an early involvement of SP in the oral carcinogenesis process.

Moreover, it has been demonstrated that malignant cells in certain types of tumors show an increased expression of NK-1R in comparison with normal cells [89–92], and overexpression of SP in tumor tissue may be associated with an increased number of NK-1R able to receive orders mediated by this neurokinin [93].

Other research has suggested some possible mechanisms by which SP and NK-1R could generate this type of effect. Thus, stimulation of NK-1R by SP activates MAPK members, including the extracellular signal-regulated kinases 1 and 2 (ERK 1/2) which are translocated into the nucleus inducing cell proliferation and protecting the cell from apoptosis [30, 36, 94, 95].

Proliferation mediated by the SP/NK-1R complex can be modulated taking into account that SP can be specifically blocked by NK-1R antagonists, such as L-773060 [89]. It has been shown that SP promotes tumor growth and that the L-773060 antagonist exerts antitumor activity against various tumor types including head and neck cancer. This antitumor activity is dose-dependent and is specifically related to the ability of antagonists to block NK-1R. These observations open new research lines for the use of NK-1R antagonists in cancer therapy [36, 89].

Tumor cell migration is an essential process for dissemination and development of metastasis, worsening the prognosis of cancer patients. It has been shown that migratory activity is a cellular function that can be modulated by neuroendocrine factors. Analyzing various cancer cell types such as MDA-MB-468 breast carcinoma, PC-3 prostate carcinoma, and SW 480 colon carcinoma cells, there have been identified both inducers for the migration activity, such as norepinephrine, dopamine, substance P, met-enkephalin, and bombesin, as well as inhibitors such as gamma-aminobutyric acid (GABA). This capacity is strongly linked to expression of other migratory markers such as high protein kinase C alpha (PKC-*α*) and low E-cadherin [96–99]. Moreover, it has been shown that using clinically established receptor antagonists, acting specific on *β*2-AR, D2 receptor, or NK-1R, respectively, the migration activity of cancer cells can be inhibited [99].

In the best of our knowledge, the findings of recent years have highlighted the importance of neuropeptides, sympathetic neurotransmitters, and also chronic stress regarding the vascularization of the tumor. This may be the basis of studies that investigate new pathways for specific inhibition of tumor cells by using existing drugs such as *β*-blockers. Given that, it is important to take into consideration that stress mediators may have other actions such as changes in the delicate mechanisms of the immune system, which may affect tumor development and tumor progression.

Moreover, it is well known that neurogenic inflammation within HNSCC favors to a large extent carcinogenesis by stimulating neurochemical changes that promote cellular proliferation. Thus, many studies are targeted towards the discovery of neuromodulators able to create a tumor-suppressing microenvironment. Such neuroactive mediators are important markers with neuromodulatory effects in skin cancers.

## 3. Role of Other Neuroendocrine Factors in HNSCC

Somatostatin (SST) is known as growth hormone release inhibitor, which has been involved in numerous other physiological events, such as regulation of exocrine and endocrine secretions, modulation of motor activity, and inhibition of gastric-stimulated gastric acid secretion.

SST plays an essential role in human tumor suppression by both direct and indirect mechanisms, for example, acting as a tumor gene suppressor with antitumor effects [100–102]. Moreover, by various mechanisms, SST has the potential to suppress tumor growth in a variety of cancers [102].

Numerous studies have attempted to highlight the aberrant expression of SST/SSTR1 proteins as a potential marker for HNSCC patients. For example, SST acts as an antitumoral agent as it inhibits growth factors, reduces vasculature, and regulates the immune system, behaving as a tumor suppressor both *in vitro* and *in vivo* [101]. Investigations regarding the role of SST and SSTR1 promoter gene hypermethylation in primary HNSCC have revealed that these epigenetic changes occur frequently in SCC cell lines and in primary tumors. Moreover, their frequency is significantly higher in patients with HNSCC and extremely low in normal fibroblasts, keratinocytes, and mucosal tissues. Regarding the method of assessing the risk of recurrence of HNSCC, this is uncertain, but the methylation of the promoter regions of neuropeptide genes in resected HNSCC is associated with tumor recurrence. Although further prospective studies are still needed to validate these assumptions in larger populations of HNSCC patients, it is important to consider these associations when designing new therapeutic regimens [20, 102].

Nitric oxide (NO) is a signaling molecule able to pass through cell and nucleus membranes and it has greater diffusion coefficient than oxygen [103, 104]. In the field of tumor biology, NO is often involved in many types of human cancers by stimulating angiogenesis and, implicitly, tumor proliferation and dissemination. Although originally considered to be only a toxic pollutant gas produced by internal combustion engines and power plants, it was discovered that NO plays the role of a biological messenger, being an endothelium-derived relaxing factor (EDRF) with significant involvement in tumor pathogenesis [105]. It has been found that NO can be a promoter of local tumor growth and metastasis by increasing neovascularization. Studies conducted by Andrade et al. [106] and Maeda et al. [107] have shown that nitric oxide synthase (NOS) inhibitors were able to reduce tumor blood flow.

NO is a highly reactive free radical synthesized by the NOS which has three isoforms: nNOS found in neural tissue, iNOS (inducible isoform) located in the basal keratinocyte layer of normal skin, and eNOS found in endothelial cells. The first and third types of NOS are expressed continuously in cells, are dependent on the level of tissue calcium, they produce NO in a pulsed manner, and are bound to the cell membrane in contrast to iNOS which is calcium independent and its expression is related to the activity of a variety of cytokines (IL-1, IFN-*γ*, and TNF-*α*) and hypoxia. However, iNOS is the most commonly associated with neoplasia. When produced by immune cells, iNOS is involved in the production of large amounts of NO with a role in pathogenic defense, cytokine production, and T helper lymphocyte expansion [103, 104, 108].

Although iNOS is normally expressed in many cells of the immune system (macrophages, T cells, and natural killer cells), it has been shown that it can be found in tumor cells also, including oral SCC [103, 104]. It has also been depicted that the high levels of iNOS are due to the malignant SCC cells and not the inflammatory cells within the stroma. In HNSCC, iNOS has been found to have intense activity in all tumor tissue with increased enzymatic expression in surrounding keratin beads [108]. Even though NO can promote adhesion of endothelial cells and vascular permeability, playing an essential role in metastasis, the whole process is not fully elucidated [109].

Recent studies showed that NO has contradictory effects, being involved in tumor progression and dissemination as well as in tumor inhibition, through direct DNA damage, inhibition of DNA synthesis, and reduction of mitochondrial activity [105, 110]. NO effects on cancer progression depend on its concentration at the site. It has been shown that mice cells produce greater levels of NO as compared to humans, possibly explaining its cytotoxic and apoptotic effect on tumors in experimental models [103, 104]. The levels of NO found in humans are usually two grades of magnitude lower than at mice; therefore, it is believed that this is the reason why, for humans, NO induces angiogenesis and favors tumor dissemination. Brennan conducted a study on patients with biopsy-confirmed oral SCC. They used isosorbide mononitrate (ISMO) as an NO donor for these patients. There were two groups, the first received placebo and the second ISMO, and one of the conclusions was that the dose of ISMO needed to induce cytotoxic levels of NO was too high, and patients started experiencing side effects [111].

Studies have shown that in HNSCC, the expression of eNOS is higher in inflammatory and neoplastic tissue compared to normal mucosa, but the levels of eNOS are reduced while the severity of dysplasia increases [110]. Moderate and severe dysplasia is correlated with high levels of iNOS which, in turn, produces consistent levels of NO that might inhibit eNOS. Increased levels of iNOS were also found in macrophages from connective tissue underlying dysplastic cells, compared to inflammatory pathologies in which less than 5% of macrophages expressed iNOS. The elevated levels of iNOS found in severe dysplasia suggest that its involvement starts early, before transformation into invasive cancer [111, 112].

Several studies have highlighted a connection between NO and VEGF in neoplastic pathology. It has been shown that the first stages of HNSCC tumor growth need high levels of NO which increases iNOS expression that facilitates angiogenesis (NO stimulates synthesis of VEGF), cellular adhesivity, and permeability [103, 104, 112]. The effect on angiogenesis was demonstrated by Gallo et al. since L-NAME (competitive inhibitor of NOS) blocks angiogenesis [104, 113]. Brennan et al. demonstrated that tumors which did not express NOS2 developed large areas of necrosis, compared to those that had high levels of NOS2, due to lack of vascular support. In addition to stimulating angiogenesis, NO also stimulates lymphangiogenesis, which facilitates lymph node metastasis [114–116].

Thorough research has highlighted the importance of angiogenesis during the progressive transformation of keratinocytes into invasive cancer. Dysplastic lesions showed abundant capillaries that resulted in vascularization in invasive SCC [117]. Further research is needed to clarify the roles of VEGF and NOS2 in the development and progression of oral cancer and to suggest possible applications of their expression for targeted therapy.

Moreover, several studies have suggested a possible correlation between NO and cyclooxygenase 2 (COX-2) in carcinogenetic processes, including HNSCC. Recent studies have shown a close association between iNOS and COX-2 activity in HNSCC and tumor angiogenesis and metastasis of lymph nodes, so iNOS and COX-2 inhibitors have been proposed as potential antitumor drugs with a strong antiangiogenic effect [116, 117].

COXs is a group of enzymes which transform arachidonic acid into prostaglandins (key mediators of inflammation) and thromboxanes. This family of enzymes consists of two isoforms: COX-1 and COX-2, the latter being the one that responds to inflammatory process. The other pathway through which arachidonic acid can be metabolized is through lipoxygenase (LOX) enzymes (5-LOX, 8-LOX, 15-LOX, and 12-LOX, the latter being implicated in tumorigenesis) inducing synthesis of leukotrienes, lipoxins, and fatty acids [118, 119].

Patients with head and neck cancer had high levels of metabolites of the enzymes mentioned above in tissue, plasma, and saliva. COX-2 promotes carcinogenesis through stimulation of angiogenesis (by modulating VEGF production), inducing metastasis and modulation of apoptosis [105]. It has been shown that stimuli like mitosis and inflammation induce COX-2 activity resulting in high levels of prostaglandins in tumor and inflammatory pathologies as compared to normal tissue, where COX-2 is undetectable. COX-2 activity in tumorigenesis is also backed by the chemopreventive effect of the nonsteroidal anti-inflammatory drugs in SCC [120, 121].

Overexpression of iNOS and COX-2 in numerous human carcinomas resulted in the release of large amounts of NO and prostaglandins (PGs), but their exact source could not be clarified because both cancer cells and stromal cells can lead to the release of metabolites and their potential pathogenic role in tumor growth remains to be fully elucidated. Being involved in biological processes such as host immune response, proliferation, and neovascularization, iNOS and COX-2 may be targets for antitumor therapy. It is known that NO has a direct effect on endothelial cells and is involved in mediating the angiogenic effect of VEGF by activating the MAPK cascade [120, 121]. However, a clear picture of the mechanism of COX-2 stimulation by NO has not yet been defined, although both are presented as important regulators of acute and chronic inflammation [122, 123].

Galanin is a neuropeptide widely expressed in the central and peripheral nervous systems and in the endocrine system. It induces growth and development of neuronal cells through binding three isoforms of galanin receptors: GALR1, GALR2, and GALR3. Many studies have shown that galanin and its receptors mediate various physiological activities in the central and peripheral nervous system, but even so the function and signaling of GALR are not fully understood in cancer cells. It is believed that GAL has an autocrine mitogenic effect in oral epithelial cells, so an anti-GAL antibody could be engineered, thus inhibiting proliferation. In recent years, expression of galanin receptors on tumors derived from epithelial cells has been thoroughly studied, aiming to develop novel therapeutic targeting [21, 124–126]. The GALR1, GALR2, and GALR3 receptors are expressed in normal and malignant keratinocytes. The proproliferative effects of GAL on human keratinocytes have suggested that they express at least one of the three receptors.

GALR1 appears to have antiproliferative effects in oral SCC. Studies have shown a significant decrease in GALR1 expression levels in oral SCC compared to normal tissue. In contrast, GALR2 has a proliferative action and it is overexpressed in HNSCC following a chromosomal translocation resulting in the loss of heterosigozity (LOH) in 18q. The loss of 18q is accompanied by GALR1 loss which is mapped to 18q23, and this is associated with an increase in the expression of another receptor, GALR2 [126]. Therefore, identifying this genetic anomaly would help elucidate the mechanism at the base of HNSCC development and progression, providing new targets for therapy. Furthermore, GAL binding to GALR1 has been demonstrated to have tumor suppressor actions, stopping proliferation and inducing apoptosis independent of p53 function [21, 54]. Therefore, the therapeutic potential of GALR expressing tumors is noteworthy, considering their independence of p53 action [54].

Unlike GALR2 and GALR3, GALR1 is a tumor suppressor. Thus, GAL is antiproliferative by GALR1 and stimulates aggressive tumor growth by GAL2 [127, 128]. Given that, GALR1 and GALR2 have antagonistic effects. In HNSCC, GALR1 expression is diminished, while GALR2 is intense. This is an issue requiring further study for GALR1 and GALR2 as targets for HNSCC therapy and exploring potential opportunities and future clinical directions.

In a recent study, the expression of neurotrophins derived from the brain was compared with neuropeptides in HNSCC, indicating that activated GALR2 induces the activated cytoplasmic T cell nuclear factor, calcineurin-2-mediated transcription (NFATC2) of cyclooxygenase-2 (COX2), which stimulates the production of prostaglandin E2 (PGE2), promoting tumor progression [129].

The dynamic interaction between nerves and cancer cells is insufficiently studied. It is assumed that there are specific biological interactions between HNSCC and nerves, as HNSCC has a tendency towards neuronal invasion, unlike other types of head and neck cancers [130]. Predicting a poor survival in HNSCC, perineural invasion is observed in up to 80% of HNSCC cases and correlates with tumor recurrence and spread [131, 132]. Despite the latest discoveries in treatment options for patients with HNSCC, survival rates have not changed significantly. Unfortunately, there is no targeted treatment since the intricate molecular mechanisms of the disease remain largely unknown. Neuronal-neoplastic synapses are specific neuronal and neuron-linked cancer cells that play an important role in metastasis [133]. Recent studies claim that GAL and GALR2 play a significant role in nerve-tumor interactions. Moreover, there are well-documented studies that highlight the spread of tumors in the brain and the brain stem through the perineural space of the nerve—the perineural spread of cutaneous SCC [134]. Although it is known that human GAL is a neuropeptide with neurotrophic and neuroprotective roles [135], it may also be involved in nonneuronal contexts, being strongly expressed in keratinocytes where it can have proliferative functions by being involved even in thermoregulation and immune response [136, 137].

In addition to proliferative effects, GAL is also involved in nociception [138]. Peripheral nerves release mediators of pain and regeneration, and GAL is one of them. It is usually expressed in a low level, but after lesions and inflammation, its level increases considerably. To support these theories, studies have been conducted that have demonstrated the induction of GALR2-mediated neuritogenesis [139, 140].

## 4. Conclusions

Given the diversity and the complexity of HNSCC, it is essential to acknowledge the molecular biology disease fundament in order to explain the mechanism of neuron-tumor interactions. Neuroendocrine factors can influence the evolution of cancer processes directly by stimulating the proliferation and tumor cell migration capacity or indirectly by decreasing the antitumor defense capacity and stimulating peritumoral angiogenesis. However, the cellular and molecular mechanisms by which neuroendocrine factors can influence the process of cutaneous-mucosal carcinogenesis are not fully elucidated and their investigation is of interest both from the perspective of fundamental sciences but especially from the perspective of clinical medicine with the identification of new diagnostic methods and therapeutic approaches in the management of cutaneous-mucosal tumors.
